# Academic Point-of-Care Manufacturing in Oral and Maxillofacial Surgery: A Retrospective Review at Gregorio Marañón University Hospital

**DOI:** 10.3390/medicina62010234

**Published:** 2026-01-22

**Authors:** Manuel Tousidonis, Gonzalo Ruiz-de-Leon, Carlos Navarro-Cuellar, Santiago Ochandiano, Jose-Ignacio Salmeron, Rocio Franco Herrera, Jose Antonio Calvo-Haro, Ruben Perez-Mañanes

**Affiliations:** 1 Department of Oral and Maxillofacial Surgery, Gregorio Marañon University Hospital, 28007 Madrid, Spain; 2 Gregorio Marañón Research Institute, 28007 Madrid, Spain; 3 Advanced Planning and 3D Manufacturing Unit (UPAM3D), Hospital General Universitario Gregorio Marañón, Dr. Esquerdo 46, 28007 Madrid, Spain; 4 Department of Surgery, Gregorio Marañon University Hospital, 28007 Madrid, Spain; 5 Department of Traumatology and Orthopedic Surgery, Gregorio Marañon University Hospital, 28007 Madrid, Spain

**Keywords:** 3D printing, point-of-care manufacturing, oral and maxillofacial surgery, biomodels, surgical guides, custom implants, hospital-based manufacturing, personalized medicine

## Abstract

*Background and Objectives:* Academic point-of-care (POC) manufacturing enables the in-hospital design and production of patient-specific medical devices within certified environments, integrating clinical practice, engineering, and translational research. This model represents a new academic ecosystem that accelerates innovation while maintaining compliance with medical device regulations. Gregorio Marañón University Hospital has established one of the first ISO 13485-certified academic manufacturing facilities in Spain, providing on-site production of anatomical models, surgical guides, and custom implants for oral and maxillofacial surgery. This study presents a retrospective review of all devices produced between April 2017 and September 2025, analyzing their typology, materials, production parameters, and clinical applications. *Materials and Methods:* A descriptive, retrospective study was conducted on 442 3D-printed medical devices fabricated for oral and maxillofacial surgical cases. Recorded variables included device classification, indication, printing technology, material type, sterilization method, working and printing times, and clinical utility. Image segmentation and design were performed using 3D Slicer and Meshmixer. Manufacturing used fused deposition modeling (FDM) and stereolithography (SLA) technologies with PLA and biocompatible resin (Biomed Clear V1). Data were analyzed descriptively. *Results:* During the eight-year period, 442 devices were manufactured. Biomodels constituted the majority (approximately 68%), followed by surgical guides (20%) and patient-specific implants (7%). Trauma and oncology were the leading clinical indications, representing 45% and 33% of all devices, respectively. The orbital region was the most frequent anatomical site. FDM accounted for 63% of the printing technologies used, and PLA was the predominant material. The mean working time per device was 3.4 h and mean printing time 12.6 h. Most devices were applied to preoperative planning (59%) or intraoperative use (35%). *Conclusions:* Academic POC manufacturing offers a sustainable, clinically integrated model for translating digital workflows and additive manufacturing into daily surgical practice. The eight-year experience of Gregorio Marañón University Hospital demonstrates how academic production units can enhance surgical precision, accelerate innovation, and ensure regulatory compliance while promoting education and translational research in healthcare.

## 1. Introduction

The integration of point-of-care (POC) manufacturing into university hospitals marks a pivotal step toward precision and truly personalized care. In this model, imaging, design, engineering, and surgical decision-making converge within the same certified clinical environment, creating an academic POC manufacturing ecosystem where assistance, education, and translational research are inseparable [1,2,3]. Rather than outsourcing, clinicians and engineers co-develop patient-specific medical devices—from anatomical biomodels to surgical guides and custom implants—inside the hospital, with immediate clinical feedback and full regulatory traceability [4,5,6,7].

Although additive manufacturing in medicine traces back to the mid-1980s—classical stereolithography was introduced by Charles Hull in 1986 and early oral and maxillofacial surgery (OMFS) applications were reported soon after—the field has matured from prototyping to regulated clinical production [8,9]. In OMFS, where three-dimensional relationships, aesthetics, and function are tightly coupled, tangible models and patient-matched devices have proven especially valuable for complex reconstructions, oncologic resections, orthognathic procedures, and trauma [10,11]. POC manufacturing extends these benefits by shortening turnaround, lowering dependency on external vendors, and giving surgeons direct control over design intent and fit [5].

Critically, POC manufacturing is not merely “3D printing at the hospital” [12,13,14,15]. It is a quality-managed production system embedded in clinical workflows. In Europe, Regulation (EU) 2017/745 (MDR) sets the overarching framework for medical devices, while in Spain Royal Decree 192/2023 operationalizes national aspects, including surveillance and traceability. Within this framework, hospitals may perform in-house manufacturing for selected Class I–IIa devices used within their own facilities; higher-risk Class IIb–III devices require manufacturer authorization and a certified quality system (e.g., ISO 13485) akin to industrial manufacturers. Implantable custom-made devices fall under the most stringent controls, with explicit limits to what can be produced entirely in-house. These requirements have steered leading academic centers to formalize governance, documentation, validation, sterilization, and post-market follow-up for every device [5,6,7,8].

Spain’s experience exemplifies this evolution. National surveys before and after the COVID-19 pandemic describe a rapid expansion of on-site 3D/AM capabilities in tertiary hospitals, with OMFS and trauma services among the most active adopters [5]. The pandemic further highlighted the resilience value of local, just-in-time manufacturing for both surgical and non-surgical needs, catalyzing institutional investment and standardization.

Within this landscape, Gregorio Marañón University Hospital (Madrid) has established a comprehensive academic POC manufacturing program integrated into the Department of Oral and Maxillofacial Surgery [5]. The unit operates under ISO 13485 and institutional governance aligned with MDR and RD 192/2023, with authorization for custom-made devices and defined pathways for sterilization and clinical use [6,7]. Beyond patient care, the unit functions as a platform for resident training and surgeon–engineer co-design, accelerating translational innovation while maintaining regulatory compliance.

Study aim. This work presents a retrospective review of the academic POC manufacturing activity for OMFS at Gregorio Marañón University Hospital from April 2017 to September 2025, encompassing 442 point-of-care manufactured devices. We (i) characterize device typologies (biomodels, surgical guides, custom implants) and clinical indications; (ii) describe materials, printing technologies, sterilization, and production metrics (working/printing time, mass); (iii) map anatomical distribution and intraoperative versus preoperative use; and (iv) discuss implementation within the European regulatory framework. Our hypothesis is that a mature academic POC ecosystem enables safe, timely, and scalable delivery of personalized devices, with measurable efficiency in surgical planning and execution and with governance structures that meet current regulatory expectations.

## 2. Materials and Methods

### 2.1. Study Design and Setting

This was an observational, descriptive, and retrospective study conducted at Gregorio Marañón University Hospital (Madrid, Spain), a tertiary academic center that integrates a certified Point-of-Care Manufacturing (POCM) unit within its clinical facilities. The study reviewed all 3D-printed medical devices and related products manufactured by the hospital’s Advanced Planning and 3D Manufacturing Unit (UPAM3D) for the Oral and Maxillofacial Surgery Department between April 2017 and September 2025. The UPAM3D operates under ISO 13485:2016 certification for quality management of medical devices and holds a European manufacturing license for Class IIb custom-made devices. Details on registry structure, variable recoding and data preprocessing are provided in Appendix A.

### 2.2. Study Population

All patients treated in the Oral and Maxillofacial Surgery Department who received a 3D-printed medical device produced through the POCM workflow during the study period were included. This encompassed patients undergoing reconstructive, oncologic, orthognathic, implantologic, and trauma-related procedures. A total of 442 workcases were recorded. Each case corresponded to at least one 3D product, including anatomical biomodels, surgical guides, or patient-specific implants. All 3D-printed devices intended for intraoperative use underwent a standardized clinical follow-up protocol. Clinical success was defined by the achievement of the preoperative plan, the accuracy of the device fit (assessed intraoperatively by the surgeon), and the absence of device-related complications. Long-term durability and performance are systematically monitored through the hospital’s Electronic Medical Record (EMR) as part of the Department’s Quality Assurance program.

### 2.3. Data Collection and Variables

Data were collected from the hospital’s internal registry and the UPAM3D digital database. Variables included the following:

- Demographics: patient age and sex.
- Clinical category: pathology type (traumatology, oncology, implantology, orthognathic surgery, instrumentation, or development).
- Device characteristics: product classification (biomodel, guide, custom implant, instrument, development prototype).
- Risk class: based on the European Medical Device Regulation (MDR 2017/745), ranging from Class I (low risk) to Class IIb (higher risk).
- Manufacturing parameters: printing technology, material type, sterilization method, print time, work hours, and print mass.
- Clinical utility: preoperative, intraoperative, or educational use.

The study excluded devices produced for other hospital departments or for research projects unrelated to oral and maxillofacial surgery.

### 2.4. Digital Workflow

While the engineering workflow involves complex software (e.g., 3D Slicer, Meshmixer), the clinical focus remains on the surgeon-led design validation, ensuring that the final device matches the intended surgical plan. The POCM process followed a standardized, traceable digital workflow integrating clinical, engineering, and regulatory stages.

1. Clinical Request: The responsible surgeon submitted a formal manufacturing request through the electronic health record system, specifying the desired device type (biomodel, cutting guide, or implant) and attaching the patient’s imaging data (CT or MRI).
2. Segmentation and Design: Imaging data were segmented using 3D Slicer (Brigham and Women’s Hospital, Boston, MA, USA), and digital modifications were performed with Autodesk Meshmixer. Designs were verified collaboratively between surgeons and biomedical engineers during a joint review meeting.
3. Approval and Documentation: Once validated, the design was approved and digitally signed by the responsible surgeon. A manufacturing prescription and traceability sheet were automatically generated according to ISO 13485 and MDR documentation standards.
4. Printing Process: Devices were printed using fused deposition modeling (FDM) (Ultimaker, Utrecht, The Netherlands) or Stereolithography (SLA) (Formlabs Form 2, Somerville, MA, USA). For metallic implants, selective laser melting (SLM) or electron beam melting (EBM) was outsourced to certified external suppliers under the hospital’s license.
5. Postprocessing and Sterilization: Printed devices underwent cleaning, curing, and inspection. Sterilization methods included ethylene oxide (55 °C), hydrogen peroxide, or saturated steam at 134 °C, depending on material compatibility. Non-sterilizable biomodels were used exclusively for planning and educational purposes.
6. Integration and Record Keeping: Each device was labeled, documented, and incorporated into the patient’s medical record. Digital files were archived for traceability and potential reprinting under controlled access.

### 2.5. Data Analysis

Descriptive statistical analysis was performed using Microsoft Excel and IBM SPSS Statistics v26 (IBM Corp., Armonk, NY, USA). Quantitative variables were expressed as means, medians, modes, and standard deviations, while categorical variables were expressed as absolute frequencies and percentages. Graphical data were represented as bar charts and histograms.

### 2.6. Ethical Considerations

This study was conducted in compliance with the ethical standards of the Declaration of Helsinki. Ethical approval was obtained from the Institutional Review Board of the Gregorio Marañón Research Institute (IiSGM). All data were anonymized, and no patient-identifiable information was used.

## 3. Results

### 3.1. Cohort and Unit of Analysis

**Figure 1:**
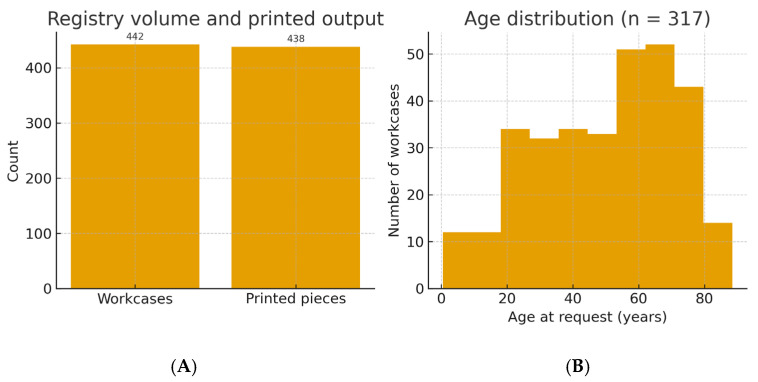
Cohort and unit of analysis for the UPAM3D registry. (**A**) Total number of point-of-care (POC) workcases (manufacturing requests) recorded between April 2017 and September 2025 (n = 442) compared with the aggregate number of printed pieces generated in the same period (n = 438), illustrating that a single workcase could yield zero, one, or multiple printed products. (**B**) Histogram of patient age at the time of the request for workcases with complete birth date data (n = 317). The age distribution shows a wide clinical spectrum, with a mean age of 49.9 years and a median of 53.5 years. Sex data were inconsistently recorded and are therefore not reported in aggregate.

Between April 2017 and September 2025, the UPAM3D registry captured 442 workcases (manufacturing requests) from the Oral and Maxillofacial Surgery Department. Because a single workcase could include zero, one, or multiple printed pieces, the period yielded an aggregate of 438 printed pieces (mean 0.99 ± 0.70 per workcase; range 0–4). Age at request was available in 317 workcases (mean 49.9 years; median 53.5). Sex data were inconsistently recorded and are therefore not reported in aggregate (Figure 1). The initial registry was designed primarily for manufacturing traceability under ISO 13485 standards, which led to a focus on production metrics over comprehensive demographic recording during the early years of the unit.

### 3.2. Intended Clinical Use

**Figure 2:**
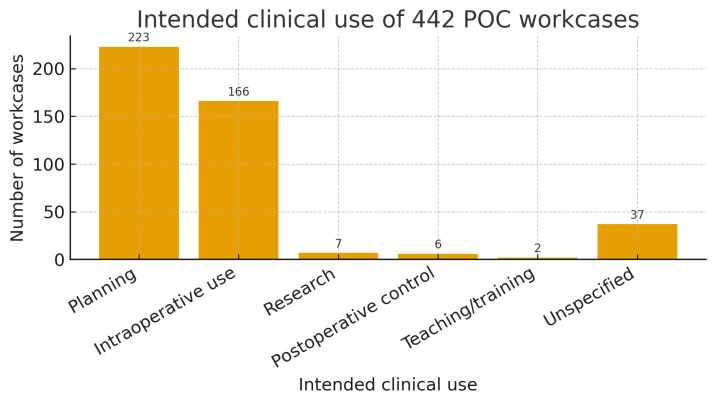
Bar chart showing the intended clinical use of 442 point-of-care (POC) workcases. The *x*-axis displays the main categories of intended use (planning, intraoperative use, research, postoperative control, teaching/training, and unspecified), and the *y*-axis indicates the number of workcases in each category. Preoperative planning was the most frequent indication (223/442, 50.5%), followed by intraoperative application (166/442, 37.6%). Research, postoperative control, and education/training accounted for a small proportion of workcases, while 8.4% of cases had an unspecified intended use.

The primary intended use was preoperative planning in 223/442 (50.5%) workcases, followed by intraoperative application in 166/442 (37.6%). Remaining indications were research (1.6%), postoperative control (1.4%), and education/training (0.5%); 8.4% were unspecified (Figure 2). Main clinical indications (trauma, oncology, implantology and orthognathic) were assigned using keyword-based text mining of free-text clinical requests (Appendix B).

### 3.3. Device Categories

**Figure 3:**
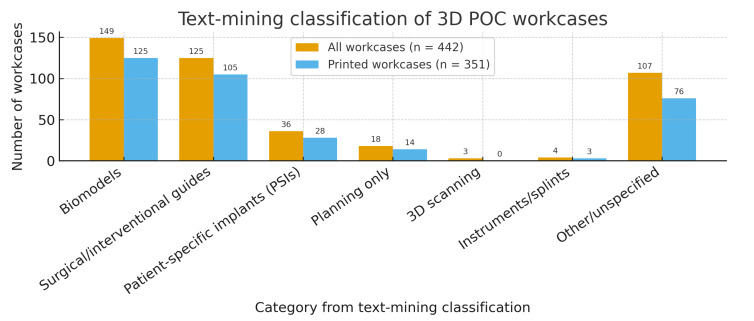
Text mining-based classification of clinical requests for point-of-care (POC)-manufactured products. Bars show the number of workcases assigned to each category for all clinical requests (dark bars; n = 442) and for the subset of workcases that actually resulted in printed pieces (light bars; n = 351). Automated classification of the free-text requests identified biomodels, surgical/interventional guides, and patient-specific implants (PSIs) as the three dominant categories, with smaller contributions from planning-only requests, 3D scanning, instruments/splints, and other/unspecified indications. For regulatory mapping within the EU MDR framework, biomodels and instruments were considered Class I devices, guides Class IIa, and PSIs Class IIb.

**Figure 4:**
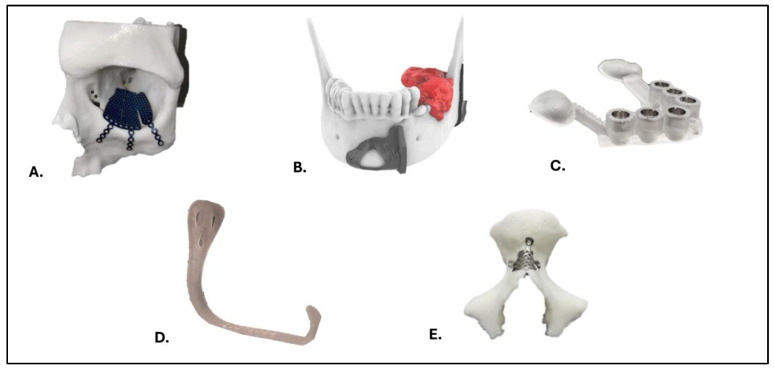
Examples of 3D-printed medical devices by classification: This image displays various types of 3D-printed medical devices used in clinical settings, organized by classification. The categories include (**A**) anatomical models (Class I), (**B**) surgical cutting guides (Class IIA), (**C**) guided implant splints (Class IIA), (**D**) surgical instruments (Class I), (**E**) patient-specific device (Class IIB). Each item is designed for specific surgical applications, reflecting the versatility of 3D printing technology in healthcare.

Text mining of the clinical requests shows that most printed products fell into three practical categories: biomodels, surgical/interventional guides, and patient-specific implants (PSIs). The keyword sets used to assign trauma, oncology, implantology and orthognathic labels are summarized in Appendix B. Considering all 442 workcases, automated classification of the free-text requests identified biomodels in 149 workcases, guides in 125, and PSIs in 36; planning-only requests accounted for 18, 3D scanning for 3, instruments/splints for 4, and other/unspecified for the remainder. Among workcases that actually yielded printed pieces (n = 351), the mix was biomodels 125, guides 105, PSIs 28, planning-only 14, instruments/splints 3, and other/unspecified 76. For regulatory mapping, biomodels/instruments were considered Class I, guides Class IIa, and PSIs Class IIb within the EU MDR framework (Figure 3 and Figure 4).

### 3.4. Technologies and Materials

**Figure 5:**
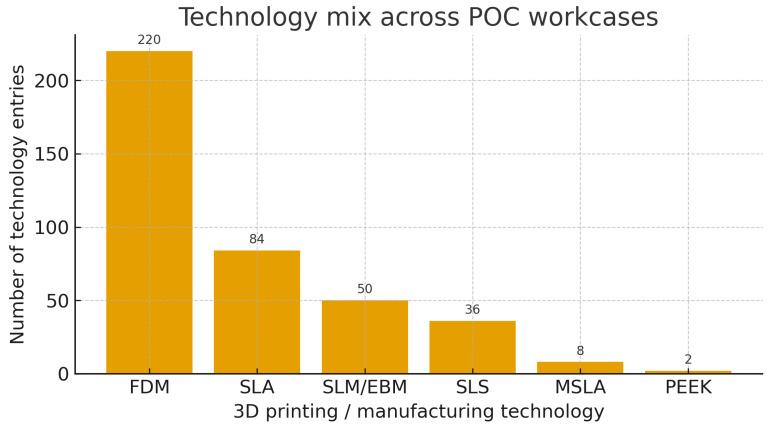
Technology mix across point-of-care (POC) workcases. Bar chart showing the number of technology entries recorded in the UPAM3D registry, acknowledging that multiple technologies could be used within a single workcase (sum across multi-technology cases = 400 entries). Fused deposition modeling (FDM) was the most frequently recorded technology (220 entries), followed by stereolithography (SLA; 84), selective laser melting/electron beam melting (SLM/EBM; 50), selective laser sintering (SLS; 36), masked SLA (MSLA; 8), and PEEK-based manufacturing (2). In-house production relied mainly on FDM and SLA, whereas SLM/EBM was reserved for metallic patient-specific implants manufactured externally under the hospital’s quality system.

Across all workcases, multiple technologies could be used per case. The technology mix was as follows: FDM 220 entries, SLA 84, SLM/EBM 50, SLS 36, MSLA 8, PEEK 2 (sum across multi-tech cases = 400 technology entries). In-house production relied mainly on FDM and SLA, while SLM/EBM was reserved for metallic PSIs via licensed external manufacturing under the hospital’s quality system (Figure 5).

**Figure 6:**
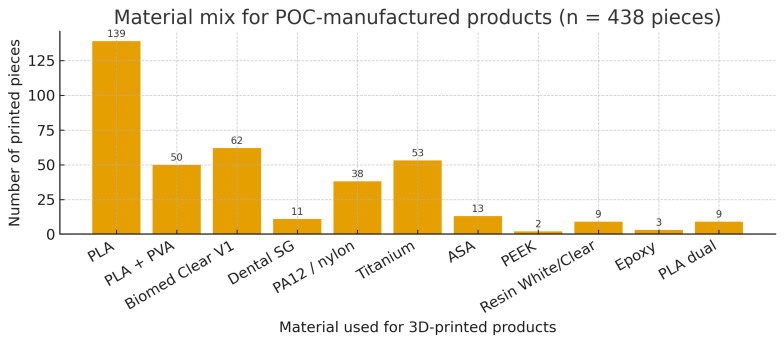
Material mix for point-of-care (POC)-manufactured products. Bar chart showing the number of printed pieces fabricated with each material, acknowledging that multiple materials could be used across the 438 printed pieces. PLA (n = 139) and PLA + PVA (n = 50) predominated for planning models. Biomed Clear V1 (n = 62) and Dental SG (n = 11) were the most frequently used certified resins, whereas PA12/nylon (n = 38) supported robust non-implantable parts and Titanium (n = 53) covered patient-specific implants (PSIs). ASA (n = 13) and PEEK (n = 2) were used selectively. Small counts were observed for Resin White/Clear (n = 9), Epoxy (n = 3), and PLA dual (n = 9).

Material selection reflected this mix: PLA (n = 139) and PLA + PVA (n = 50) predominated for planning models; Biomed Clear V1 (n = 62) and Dental SG (n = 11) were the most frequent certified resins; PA12/nylon (n = 38) supported robust non-implantable parts; Titanium (n = 53) covered PSIs; ASA (n = 13) and PEEK (n = 2) were used selectively; small counts appeared for Resin White/Clear, Epoxy, and PLA dual (Figure 6).

### 3.5. Sterilization

Sterilization methods (selected by material compatibility and intended use) were hydrogen peroxide plasma in 281/442 (63.6%) workcases and saturated steam 134 °C in 94/442 (21.3%); non-sterilizable items (planning/education) represented 15/442 (3.4%). In 52/442 (11.8%), the method was not specified in the registry.

### 3.6. Anatomical Distribution and Clinical Focus

Keyword analysis of the clinical indications showed prominent involvement of the orbit (≈87 workcases)—chiefly trauma—followed by mandible (≈122) and maxilla (≈58); additional sites included cranium (≈17), TMJ/condyle (≈10), frontal bone (≈13), and nasal complex/zygoma (≈3 each). The pattern aligns with our service mix: trauma and oncology dominate the requests, with orthognathic and implantology representing smaller but relevant clusters.

**Figure 7:**
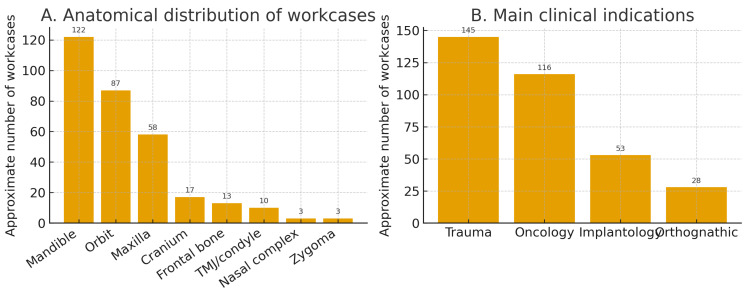
Anatomical distribution and main clinical indications of point-of-care (POC) workcases. (**A**) Approximate anatomical distribution of workcases based on keyword analysis of the clinical indications, showing prominent involvement of the mandible (≈122 workcases), orbit (≈87), and maxilla (≈58), with additional cases affecting the cranium, frontal bone, temporomandibular joint/condyle, and nasal complex/zygoma. (**B**) Broader classification of indications using enriched keyword sets, highlighting that trauma (≈145 workcases) and oncology (≈116) dominate the case mix, whereas implantology (≈53) and orthognathic surgery (≈28) represent smaller but clinically relevant clusters. Overlaps between categories are expected, as some workcases legitimately fall into more than one indication.

A broader classification of indications using enriched keyword sets identified trauma in ~145 workcases, oncology in ~116, implantology in ~53, and orthognathic in ~28, recognizing that some workcases legitimately overlap categories (e.g., oncologic resection with post-traumatic sequelae) (Figure 7).

### 3.7. Manufacturing Effort and Throughput

**Figure 8:**
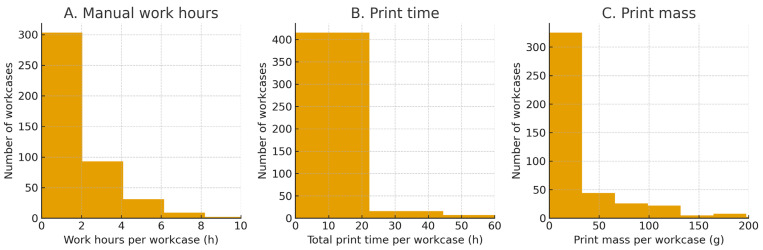
Work hours, print time, and print mass for point-of-care (POC)-manufactured workcases. (**A**) Distribution of manual work hours per workcase (total 907 h; mean 2.05 ± 2.93 h; range 0–41 h). (**B**) Distribution of total print time per workcase (cumulative 3315 h; mean 7.50 ± 23.86 h; range 0–445 h). (**C**) Distribution of print mass per workcase (total 14,362 g; mean 32.5 ± 75.1 g; range 0–659 g). To improve visualization of the central distribution and median values, the x-axes are truncated at 10 h (**A**), 60 h (**B**), and 200 g (**C**); a small number of large, complex biomodels lies beyond these limits, reflecting the right-skewed nature of all three distributions.

The total work hours across the series were 907 h (mean 2.05 ± 2.93 h per workcase; range 0–41 h). Cumulative print time reached 3315 h (mean 7.50 ± 23.86 h; range 0–445 h). The total print mass was 14,362 g (mean 32.5 ± 75.1 g; range 0–659 g). These distributions are right-skewed, reflecting a minority of large, complex biomodels that consume disproportionate printing resources (Figure 8).

**Figure 9:**
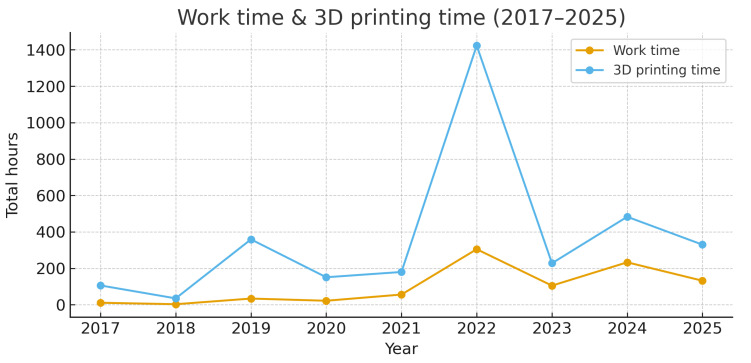
Annual distribution of manual work time and 3D printing time for point-of-care (POC) manufacturing projects in the UPAM3D registry between 2017 and 2025. The blue line represents the cumulative work time (in hours) spent on case preparation and design per year, whereas the orange line represents the cumulative 3D printing time per year.

Clinical Implications of Manufacturing Metrics: The results indicate that the design phase (mean 3.4 h) remains the primary bottleneck requiring surgeon involvement, whereas the printing time (mean 12.6 h) is an automated background process. This distinction is crucial for hospitals planning to integrate POCM into routine surgical schedules. Annual cumulative work time and 3D printing time did not increase linearly over the study period (Figure 9). Early years show low cumulative values, followed by a marked peak in 2022 and stable or slightly decreasing totals thereafter despite sustained activity.

### 3.8. Technical Standards for Technology and Material Selection

Based on our retrospective analysis, we have established standardized protocols for technology selection based on clinical intent. For anatomical biomodels used in preoperative planning, fused deposition modeling (FDM) with PLA filaments remains the standard due to its cost-effectiveness. When the application requires intraoperative use, such as surgical cutting guides, Stereolithography (SLA) or Digital Light Processing (DLP) is preferred due to the availability of autoclavable, biocompatible resins.

Regarding definitive patient-specific implants (PSIs), although medical-grade titanium remains the predominant material in our specialty for its superior mechanical properties and osteointegration, our current model relies on the outsourcing of titanium manufacturing. This strategic decision ensures maximum efficiency and regulatory compliance, as external certified partners provide the high-energy metal sintering (SLM/DMLS) infrastructure required. For in-house manufactured implants, our workflow focuses on high-performance polymers like Polyamide (via SLS) or PEEK (via FDM), reserved for specific reconstructive cases where plastic-based materials are indicated.

### 3.9. Temporal Trends in Model Utilization

**Figure 10:**
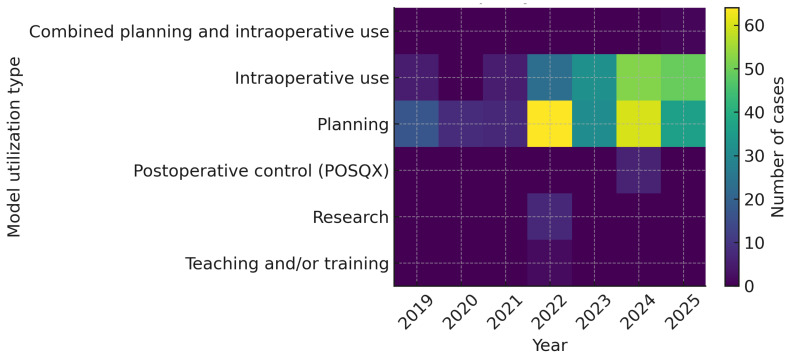
Heatmap showing the annual distribution of point-of-care (POC) manufactured cases according to model utilization type between 2019 and 2025. The *x*-axis represents the year of registration, and the *y*-axis shows the intended use of the model (planning, intraoperative use, postoperative control (POSQX), research, teaching and/or training, and combined planning and intraoperative use). Color intensity indicates the number of cases in each category. A total of 442 POC cases were included; only cases with complete data on utilization type are represented in the heatmap.

In the early years of the hospital FabLab (2014–2017), tangible 3D prints were frequently produced for patient/family communication, teaching/training, and simulation. As the program matured and clinical pathways standardized to POCM (2017–2025), utilization progressively shifted toward practical, care-embedded applications. Since 2023, communication and teaching are no longer based on printed models (we rely on digital visualization at point of care), and simulation prints are reserved only for very complex cases or for academic/research purposes. In parallel, requests for planning and intraoperative use—including guide-based procedures and PSI workflows—became the dominant indications, with a growing number of cases combining both uses within the same episode (Table 1 and Figure 10).

## 4. Discussion

Our registry of 442 workcases supports the working hypothesis that an academic point-of-care manufacturing (POCM) model embedded in a university hospital can deliver clinically relevant value across CMF indications, with the greatest demand concentrated in preoperative planning and intraoperative guidance [15,16,17,18,19,20]. These patterns mirror our hospital’s initial, cross-departmental experience, which documented high volumes of anatomical models, the progressive adoption of biocompatible resins for guides, and selective co-design of patient-specific implants with certified partners [21,22,23,24]. Together, both datasets depict a maturation from “hospital 3D printing” toward regulated POCM with mixed in-house/outsourced pathways (Table 2).

### 4.1. Interpretation Versus Prior Evidence and Our Hypotheses

We hypothesized that CMF would be a disproportionate beneficiary of academic POCM due to the region’s anatomical density and the premium on occlusion, symmetry, and margin control [25,26,27,28]. Our results confirm that biomodels dominate for trauma (notably orbit), and guides are pivotal in oncologic mandible and orthognathic workflows—exactly the use-case triad highlighted in the broader, hospital-wide report (planning models, intraoperative guides, and PSI co-design) [29,30,31,32]. The earlier study also showed that segmentation effort, not only device design, drives staff time—explaining why complex biomodels can equal or exceed guides in working hours—an observation reproduced in our series [33,34,35].

Externally, national data indicate Spain’s shift toward mixed models (in-house for polymers/resins and outsourcing for some services, such as metallic PSI), the rise in centralized request systems, and the formalization of bioengineer–surgeon–radiologist teams [5,33,36,37,38,39]. Our CMF program conforms to this picture, suggesting that our findings are generalizable to other academic centers developing POCM under similar organizational constraints [5,32,33,39].

### 4.2. Broader Institutional and Healthcare Context

Our prior institutional paper positioned the university hospital as a POC hub capable of rapid iteration, documented reductions in lead time/costs, and described quality governance (ISO 13485) as essential for scale [5]. The present CMF-focused expansion fits that trajectory: polymer/resin devices are produced in-house; implantables follow a licensed Class IIb route with industry partners, preserving traceability and biovigilance under EU MDR 2017/745 and Spanish RD 192/2023 [6,7,33]. This dual pathway aligns with national adoption surveys forecasting stronger in-house labs coupled to selective outsourcing as the practical standard of care. Selection Criteria for Point-of-Care Technologies: Choosing the right technology is a balance between clinical demand and resource availability. For institutions starting a POCM unit, we recommend SLA/DLP for applications requiring high precision and biocompatibility (e.g., cutting guides), while FDM remains the most cost-effective solution for non-sterile anatomical models and preoperative planning.

### 4.3. Clinical Implications

For orbital trauma, mirrored models and pre-bent meshes help restore volume/symmetry and reduce intraoperative adjustment—use cases explicitly emphasized in our hospital’s earlier multi-specialty program. In mandibular oncology, cutting/drilling guides facilitate margin control and reconstructive accuracy; in orthognathic surgery, occlusal splints and rehearsal models translate virtual plans to accurate skeletal movements. These application domains reflect where POCM delivers the highest GIRFT-style predictability (getting it right first time) and are consistent with the institutional argument that POCM increases efficiency across the treatment continuum [40,41,42].

### 4.4. Temporal Trends and Scalability of POC Manufacturing

Annual cumulative work time and 3D printing time did not increase linearly over the study period (Figure 9). Early years (2017–2019) reflect the implementation phase of the POC program with a limited number of pilot cases and incomplete registry capture. The COVID-19 pandemic (2020–2021) reduced elective case volume, concentrating activity on oncologic and urgent cases. A marked peak in 2022 corresponds to the post-pandemic rebound and full integration of UPAM3D workflows into routine care. Thereafter, total annual hours remained stable or slightly decreased despite a high number of workcases, consistent with progressive gains in efficiency (standardized templates, faster printers and more experienced staff) and a shift towards smaller, guide-focused cases. Overall, these trends suggest that the service has scaled up while reducing the average work and printing time per case [30,32,43].

### 4.5. Strengths and Limitations of the Academic POCM Model

#### 4.5.1. Strengths

- Deep personalization and anatomical fidelity. Patient-specific biomodels, cutting/drilling guides, and PSI translate virtual plans into reproducible intraoperative actions. Surgeon–engineer co-design formalizes surgical intent (osteotomies, resection margins, implant seating, occlusal targets) with explicit tolerances and offsets, improving fit, alignment, and predictability across CMF indications (orbit, mandible, orthognathic) [44,45]. From a clinical standpoint, this high fidelity allows the surgeon to rehearse complex maneuvers on an exact physical replica of the patient’s anatomy, shifting the focus from technical troubleshooting to surgical precision and safety before the first incision is made.
- Operating room efficiency and time savings. Pre-bent meshes/plates, guide-based osteotomies, and rehearsed sequences reduce intraoperative decision time and instrument changes, shortening OR time and anesthesia exposure and improving team throughput. Surgeon–engineer co-design formalizes surgical intent. While engineering parameters like tolerances and offsets are managed in the background, the clinical output is a device that fits the patient’s specific bone morphology perfectly. This synergy ensures that the final product is not just an engineering success, but a clinically optimized tool that reduces intraoperative “trial and error”.
- Fewer complications and reinterventions. More accurate osteotomies and restored volumes lower malposition, step-offs, and asymmetry, reducing revision surgery and unplanned imaging; guide-assisted drilling/placement mitigates iatrogenic error.
- System-level efficiency and cost containment. Shorter OR time, fewer returns to theater, and reduced implant wastage generate direct cost savings; earlier functional recovery can shorten length of stay and downstream rehabilitation needs. POCM also minimizes outsourcing lead-times and logistics by enabling just-in-time manufacturing.
- Patient safety and risk control. ISO-13485 design controls, material-appropriate sterilization, and device release checklists embed safety throughout the workflow. Physical models enhance team briefings and informed consent. Full traceability supports post-market surveillance and incident learning.
- Academic value and workforce development. The university setting structures training (surgeons in segmentation/VSP; engineers in clinical constraints), accelerates translational research, and standardizes quality metrics for cross-service benchmarking [5,33,36,43].
- Summary for the Clinician: Beyond the technical specifications of printers and software, the primary strength of this POCM model is the reduction in surgical uncertainty. By integrating manufacturing into the clinical team, we transform complex digital data into tangible surgical confidence, directly impacting operative speed and patient safety.

#### 4.5.2. Limitations and Operational Frictions

- Time-to-device in urgent polytrauma, especially for large biomodels requiring long print/curing cycles.
- Data completeness: operational registries may lack full demographics or granular outcomes, limiting adjusted analyses.
- Cost, reimbursement, and regulatory overhead, repeatedly cited as barriers to diffusion and long-term sustainability.
- Prescriber readiness: surgeons must be trained in segmentation/VSP and conversant with biomedical–engineering terminology (tolerances, offsets, STL/CAD conventions, sterilization limits); low literacy increases rework and lead-times.
- Talent dynamics: difficulty attracting and retaining biomedical engineers; turnover causes loss of tacit knowledge and recurrent onboarding with measurable productivity dips.
- Triage under exponential demand: as multi-specialty utilization grows, prioritization becomes complex; competing requests require formal governance (queues, urgency scores, cut-off times) to avoid bottlenecks and inequities.
- Change management and heterogeneous adoption: a small group of early adopters drives demand, while many clinicians show slow uptake due to workflow disruption, learning curve, or uncertain perceived value, hindering uniform standard-of-care adoption.
- Interoperability and process variance: heterogeneous software stacks (open-source vs. certified) and mixed in-house/outsourced routes (e.g., SLM/EBM for PSI) introduce variability in timelines and documentation burden.

In synthesis, academic POCM delivers meaningful gains in personalization, surgical efficiency, complication reduction, cost containment, and patient safety, but remains management-intensive. Success depends on structured training, talent retention, transparent prioritization, and rigorous regulatory compliance to achieve equitable, hospital-wide adoption.

### 4.6. Limitations and Future Research Directions

#### 4.6.1. Limitations

This study has several limitations. First, it is a single-center experience from a high-volume academic hospital with an established POCM program; therefore, generalizability to smaller centers or institutions at earlier stages of adoption may be limited. Second, the retrospective design relies on operational registries with incomplete demographic fields, which constrains adjusted analyses and may introduce information bias. Third, although the hospital operates a multi-specialty POCM unit, the present analysis is limited to one clinical specialty (CMF); thus, findings may not extrapolate to services with different case mixes, regulatory pathways, or device portfolios. Finally, the outcomes reported are predominantly process and utilization metrics; comparative clinical endpoints and validated patient-reported outcome measures (PROMs) were not prospectively collected. Accordingly, future prospective studies should link manufacturing and operational performance indicators to standardized short- and long-term clinical outcomes and patient quality of life. While long-term PROMs were beyond the scope of this operational evaluation, the high rate of surgical plan fulfillment and the absence of device-related reoperations in our cohort support the feasibility and operational robustness of the POCM model in maxillofacial reconstruction.

#### 4.6.2. Future Research Directions

To address these gaps and to build generalizable evidence under MDR-compliant frameworks, we propose the following:

1. Comparative effectiveness. Multicenter, prospective studies in orbital fractures, mandibular reconstruction, and bimaxillary orthognathic surgery comparing POCM-enabled workflows versus conventional care on operative time, revision rates, symmetry/occlusion metrics, complications, and PROMs.
2. Health economics. Cost–utility analyses comparing academic POCM with outsourced/industry pathways, explicitly modeling capital and personnel costs, material/sterilization expenses, and opportunity cost of OR time saved.
3. Process science and quality. Cross-site benchmarking of design controls, device release criteria, material-specific sterilization strategies, and failure modes (VSP/printing/postprocessing), to define shared KPIs and maturity indices for hospital POCM units.
4. Technology maturation. Validation of AI-assisted segmentation, multi-material printing for CMF use, and faster certified resins suitable for intraoperative guides to shorten design-to-delivery cycles and reduce variability.
5. Network models. Prospective evaluation of hub-and-spoke POC networks and centralized prioritization committees to test whether mixed in-house/outsourced production improves equity of access, scalability, and time-to-device across regions.

In summary, acknowledging its single-center, retrospective nature and specialty focus, our series—interpreted alongside the hospital’s multi-specialty experience and national adoption data—supports academic POCM as a scalable, quality-governed paradigm for patient-specific CMF care. The next step is coordinated, multicenter research that quantifies clinical and economic impact and standardizes processes across institutions.

## 5. Conclusions

In this single-center registry of 442 workcases, an academic point-of-care manufacturing (POCM) model proved feasible and safe for routine craniomaxillofacial (CMF) care. Demand was led by patient-specific biomodels for trauma—especially orbit—and by guides and PSI supporting oncologic mandibular reconstruction and orthognathic workflows. The program has evolved from early, education-oriented printing toward predominantly clinical applications integrated into planning and intraoperative phases.

Across indications, POCM delivered the core advantages expected of personalized surgery: greater anatomical fidelity and plan-to-execution accuracy, shorter operating time, and potential reductions in complications, reinterventions, and length of stay, while embedding safety through ISO-13485 design controls, material-appropriate sterilization, and full traceability. These benefits were enabled by co-located surgeon–engineer teams and standardized co-design and release processes.

However, meaningful constraints remain—retrospective, single-center design, specialty focus, and operational challenges common to hospital labs (time-to-device in urgent polytrauma, cost/reimbursement, prescriber training, talent retention, and prioritization across services). Consequently, generalizability to smaller or less experienced centers should be made with caution.

Future work should move beyond descriptive series toward multicenter prospective comparisons and health-economic evaluations, alongside process standardization (design controls, sterilization by material, KPIs) under MDR-compliant governance. In parallel, hub-and-spoke academic networks, validated AI-assisted segmentation, and faster certified materials may compress design-to-delivery cycles and broaden equitable access.

Overall, our experience supports academic POCM as a scalable, quality-governed paradigm for patient-specific CMF surgery that improves efficiency and safeguards patients, while laying a practical path for evidence-based adoption at the health-system level.

## Figures and Tables

**Table 1:** Distribution of required products for each product utility (percentage of workcases with both fields available, n = 291).

Required Product	Communication	Teaching	Instrumental	Research	Intraoperative Utility	Preoperative Planning	Others	Total (%)
Surgical guide/interventional procedure	0.00	0.00	0.00	1.03	29.21	0.00	0.00	30.24
Anatomical model	0.00	0.34	0.00	0.00	0.34	68.73	0.00	69.42
Navigation	0.00	0.00	0.00	0.00	0.00	0.34	0.00	0.34
Total (%)	0.00	0.34	0.00	1.03	29.55	69.07	0.00	100.00

Percentages are calculated over the 291 workcases in which both required product and product utility were specified in the UPAM3D registry. “Surgical guide/interventional procedure” includes both standard surgical guides and Class IIa guides; “Anatomical model” corresponds to 3D-printed models; “Navigation” corresponds to navigation tools. Communication and instrumental uses were not explicitly coded in this dataset, so their percentages are 0 in this table.

**Table 2:** Technical details of the 3D printing projects.

	Summary	Procedure				Anatomical Model				Navigation			Total			
		3D Model	DICOM	Other	Total	3D Model	DICOM	Other	Total	3D Model	DICOM	Total	3D Model	DICOM	Other	Total
Work time (hours)	Mean	2	3.55	4.5	3.45	2.33	3.02	0.67	2.97		2	2	2.11	3.16	2.2	3.11
	Median	1	3	4.5	3	3	2	0	2		2	2	1	2	2	2
3D printing time (hours)	Mean	3.17	4.85	7.5	4.79	30.67	14.69	18	15.01		0	0	12.33	11.93	13.8	11.98
	Median	3	2	7.5	2	21	9	10	10		0	0	4	6	10	6
Quantity 3D printing material (grams)	Mean	19.67	14.91	0	14.89	212.33	52.15	412.33	60.73		0	0	83.89	41.71	247.4	47.17
	Median	19.5	5	0	5	80	30	460	30.5		0	0	25	6	118	13

Technical details of point-of-care (POC) 3D-printing projects, stratified by required product and type of input material. “Procedure” includes both standard surgical guides and Class IIa guides; “Anatomical model” corresponds to 3D-printed anatomical models; “Navigation” corresponds to navigation tools. Input material was classified as 3D model (STL or equivalent), DICOM data, or “Other” (e.g., external files or mixed inputs). Values are means and medians of manual work time, 3D printing time, and printed mass for workcases that both yielded printed pieces and had the required product and input material specified (n = 258). The rightmost column summarizes overall values across all product and input combinations.

## Data Availability

The authors declare that they have followed their center’s protocols on the publication of patient data. All data analyzed during the current study are available from the corresponding author on reasonable request.

## Abbreviations

The following abbreviations are used in this manuscript:

POC	Point-of-care
POCM	Point-of-care manufacturing
CMF	Craniomaxillofacial
OMFS	Oral and Maxillofacial Surgery
UPAM3D	Advanced Planning and 3D Manufacturing Unit
IiSGM	Gregorio Marañón Research Institute
ISO	International Organization for Standardization
ISO 13485	ISO standard for medical device quality management systems
EU	European Union
MDR	Medical Device Regulation (Regulation (EU) 2017/745)
RD 192/2023	Royal Decree 192/2023 (Spanish regulation for medical devices)
AEMPS	Spanish Agency of Medicines and Medical Devices
QMS	Quality Management System
IRB	Institutional Review Board
EHR	Electronic Health Record
VSP	Virtual Surgical Planning
PSI	Patient-Specific Implant
CT	Computed Tomography
MRI	Magnetic Resonance Imaging
DICOM	Digital Imaging and Communications in Medicine
CAD	Computer-Aided Design
STL	Stereolithography file format
FDM	Fused Deposition Modeling
SLA	Stereolithography
MSLA	Masked Stereolithography
SLS	Selective Laser Sintering
SLM	Selective Laser Melting
EBM	Electron Beam Melting
PLA	Polylactic Acid
PVA	Polyvinyl Alcohol
PA12	Polyamide-12 (nylon-12)
PEEK	Polyether-ether-ketone
H_2_O_2_	Hydrogen peroxide (plasma sterilization)
EO	Ethylene oxide (sterilization)
IFU	Instructions for Use
OR	Operating Room
LOS	Length of Stay
PROMs	Patient-Reported Outcome Measures
KPI(s)	Key Performance Indicator(s)
TMJ	Temporomandibular Joint
GIRFT	Getting It Right First Time (service-improvement approach)
POSQX	Postoperative control (label used in utilization heatmap)

## Appendix A

### Appendix A.1. Registry Structure and Inclusion Criteria

The UPAM3D registry is a prospectively maintained database that records all manufacturing requests (workcases) processed by the hospital-based point-of-care (POC) 3D-printing unit. Each row corresponds to a single workcase, which may generate zero, one or multiple printed pieces.

For the present analysis, we exported all entries from April 2017 to September 2025 corresponding to requests initiated by the Oral and Maxillofacial Surgery Department. After removing test entries and non-clinical administrative records, the final dataset comprised 442 workcases, which yielded an aggregate of 438 printed pieces.

The original Excel file contains general identifiers (case code, registry number), clinical metadata (referring clinician, service, brief indication), manufacturing metadata (requested product, technology, materials, sterilization method, dates) and workload metrics (work hours, print time, print mass).

Only variables relevant to the present study were used and, when necessary, recoded into standardized analytical categories as described below. Workcases with obviously erroneous dates or negative durations were checked against the original database and amended or excluded as appropriate.

### Appendix A.2. Derived Variables and Recoding Strategy

Several analytical variables used in the main text were derived from raw Excel fields through recoding or aggregation:

- Year of request was obtained from the Registration date field and used for temporal analyses.
- Required product was derived from the free-text field Request type and grouped into Anatomical model, Surgical guide/interventional procedure, Patient-specific implant (PSI), Planning-only (3D study without printing), 3D scan, Instruments/splints, Navigation, and Other/unspecified. “Surgical guide/interventional procedure” includes both standard guides and Class IIa guides.
- Intended clinical use was derived from Model utility and recoded as Preoperative planning, Intraoperative use, Research, Teaching/training, Postoperative control (POSQX), and Unspecified. Mixed entries labeled “Intraoperative planning and utility” were counted under Intraoperative use for descriptive summaries.
- Printed workcase (yes/no) was defined as any workcase with a non-zero value in at least one of the following fields: Number of printed pieces, Total printing time [h], or Total printing mass [g/mL].
- Work time (hours) captures manual time spent on segmentation, design, file preparation and related tasks.
- Three-dimensional printing time (hours) represents the sum of all print jobs associated with a given workcase.
- Print mass (grams) corresponds reflects the total mass of printed material per workcase.
- Technology group may include multiple entries per workcase. Technologies were grouped as FDM, SLA, SLM/EBM, SLS, MSLA, PEEK and Other. The technology counts reported in the main text refer to the total number of technology entries (multi-technology workcases contributing more than once).
- Material group can list several materials per workcase. Materials were grouped into PLA, PLA + PVA, Biomed Clear V1, Dental SG, PA12/nylon, Titanium, ASA, PEEK, Resin White/Clear, Epoxy, PLA dual, and Other. Material counts reflect the number of material entries across workcases.
- Patient age at request (years) was computed for workcases with both Registration Date and Birth Date available, by dividing the difference in days by 365.25. Age was used only in aggregate (mean, median and histograms).
- Anatomical site and main clinical indication (trauma, oncology, implantology, orthognathic) were obtained through keyword analysis of the free-text indication fields (Description of the need, Description by the worker). A workcase could be assigned to more than one indication category when clinically appropriate (e.g., oncologic resection with post-traumatic sequelae).

**Table A1:** This table summarizes the key variables extracted or derived from the UPAM3D registry for this study.

Excel Field (Original)	Derived Variable Used in this Study	Type	Operational Definition/Coding Rule
Registration date	Year of request	Integer	Calendar year extracted from the registration date.
Case/Registry number	Workcase ID	Identifier	Unique identifier for each manufacturing request.
Request type	Required product	Categorical	Recoded into anatomical model, surgical guide/interventional procedure, patient-specific implant (PSI), planning-only, 3D scan, instruments/splints, navigation, or other/unspecified.
Model utility	Intended clinical use	Categorical	Recoded into preoperative planning, intraoperative use, research, teaching/training, postoperative control (POSQX), or unspecified. Mixed “planning + intraoperative” labeled as intraoperative use.
Input data type	Input data type	Categorical	Recoded into 3D model (STL or equivalent), DICOM, or other (mixed/alternative inputs).
Technology	Technology group	Categorical	Grouped as FDM, SLA, SLM/EBM, SLS, MSLA, PEEK, other. Multi-technology workcases contribute one count per technology used.
Printing material	Material group	Categorical	Grouped as PLA, PLA + PVA, Biomed Clear V1, Dental SG, PA12/nylon, Titanium, ASA, PEEK, Resin White/Clear, Epoxy, PLA dual, or other. Multi-material workcases contribute one count per material used.
Number of printed pieces	Number of printed pieces	Numeric	Total number of individual 3D-printed parts produced for the workcase.
Total print mass [g/mL]	Print mass (grams)	Numeric	Total mass of printed material per workcase. Used to describe material consumption and skewness of workload.
Total print time [h]	3D printing time (hours)	Numeric	Sum of all print jobs (in hours) associated with the workcase.
Work hours	Work time (hours)	Numeric	Manual time spent on segmentation, design, file preparation and related tasks for each workcase.
Date of birth	Age at request (years)	Numeric	(Registration Date–Birth Date)/365.25 for workcases with both dates available; not imputed when missing.
Clinical indication description (clinician/operator)	Anatomical site	Categorical	Derived via keyword search (e.g., mandible, maxilla, orbit, cranium, TMJ/condyle, frontal bone, nasal complex/zygoma).
Free-text description fields used for text mining	Main clinical indication	Categorical	Derived via enriched keyword sets; workcases classified as trauma, oncology, implantology, orthognathic; overlaps allowed.
Printed-output fields (pieces, time, mass)	Printed workcase (yes/no)	Binary	Classified as “printed” if any of the three fields is non-zero; otherwise considered a planning-only workcase.

## Appendix B

### Keyword-Based Classification of Main Clinical Indications: Rationale and Preprocessing

To characterize the clinical focus of each workcase, we performed a simple text mining procedure on the free-text indication fields (Descripción de la necesidad, Descripción por el trabajador). The goal was to assign each workcase to one or more main clinical indication categories:

- Trauma
- Oncology
- Implantology
- Orthognathic

Because the same patient may present combined problems (e.g., post-traumatic defects reconstructed for oncologic reasons), the algorithm allowed overlapping categories (non-mutually exclusive labels).

For preprocessing, all text fields were converted to lowercase and stripped of diacritics (e.g., “ortognática” → “ortognatica”). Punctuation was removed and simple keyword searches were performed using predefined lists for each indication. When any of the keywords listed in Table A2 was present in either indication field, the corresponding category was assigned to that workcase. If multiple keyword sets were matched, multiple labels were assigned. Workcases with no relevant keywords were left unlabeled for this analysis.

The resulting classification yielded approximately 145 trauma-related workcases, 116 oncology-related workcases, 53 implantology-related workcases, and 28 orthognathic-related workcases, recognizing that some workcases contributed to more than one category.

**Table A2:** Keyword sets used to classify workcases into main clinical indication categories.

Indication Category	Example Keywords Searched	Typical Anatomical Targets/Examples	Notes on Overlaps
Trauma	“trauma”, “fracture”, “orbital fracture”, “blow-out”, “blow out”, “mandibular fracture”, “maxillary fracture”, “zygomatic fracture”, “orbital floor”, “naso-orbito-ethmoidal fracture”, “NOE”	Orbital fractures, midface and zygomatic fractures, mandibular fractures, cranial vault trauma	Trauma label is assigned whenever acute or post-traumatic defects are mentioned, regardless of concomitant oncologic or reconstructive context.
Oncology	“cancer”, “tumour”, “tumor”, “tumoral”, “oncologic”, “carcinoma”, “squamous cell carcinoma”, “SCC”, “sarcoma”, “ameloblastoma”, “osteosarcoma”, “metastasis”, “malignant lesion”	Resective surgery for oral cavity, mandible, maxilla, skull base or soft-tissue malignancies	Workcases involving reconstruction of post-oncologic defects are labeled as oncology even if a traumatic or orthognathic component is also present.
Implantology	“implant”, “implants”, “dental implant”, “zygomatic implants”, “guided implant”, “implant guide”, “guided splint”, “immediate loading”	Static/dynamic guided implant surgery, zygomatic implants, implant-supported prostheses	Implantology label is added when guided implant placement or implant-supported rehabilitation are a primary indication, including in oncologic or trauma cases.
Orthognathic	“orthognathic”, “Class III”, “Class II”, “osteotomy”, “Le Fort”, “BSSO”, “mandibular advancement”, “mandibular setback”, “maxillary advancement”	Bimaxillary orthognathic surgery, single-jaw osteotomies, segmental osteotomies	Orthognathic label is assigned regardless of concurrent minor trauma or implant placement if the primary goal is correction of dentofacial deformity.
